# Patient and public involvement in research: from tokenistic box ticking to valued team members

**DOI:** 10.1186/s12916-020-01544-7

**Published:** 2020-04-13

**Authors:** Tracy Jackson, Hilary Pinnock, Su May Liew, Elsie Horne, Elisabeth Ehrlich, Olivia Fulton, Allison Worth, Aziz Sheikh, Anna De Simoni

**Affiliations:** 1 Asthma UK Centre for Applied Research, University of Edinburgh, Edinburgh, UK; 2 Usher Institute, University of Edinburgh, Edinburgh, UK; 3 NIHR Global Health Research Unit on Respiratory Health, University of Edinburgh, Edinburgh, UK; 4 Department of Primary Care Medicine, University of Malaya, Kuala Lumpur, Malaysia; 5 Asthma UK Centre for Applied Research, Queen Mary University of London, London, UK; 6 Department of Public Health and Primary Care, Queen Mary University of London, London, UK

**Keywords:** Patient and public involvement, Community engagement, Research, Health

## Abstract

**Background:**

Patient and public involvement (PPI) in research envisages a relationship built throughout the lifespan of a research project between academics, clinicians and PPI colleagues in order to inform, plan, execute and, in due course, disseminate and translate research. To be meaningful, all stakeholders need to actively engage in this exchange of expertise. However, despite some funders requiring PPI plans to be included in grant applications, there remains a gap between what is expected and what is delivered.

**Main body:**

As an exemplar, we reflect on how, in the Asthma UK Centre for Applied Research (AUKCAR), we set out to create a supportive, organised environment with the overarching value of ‘keeping patients at the heart of everything we do’. The key has been in planning and creating a suitably funded organisational infrastructure with dedicated PPI researchers along with the development of and expectation to abide by an agreed set of norms and values. Specifically, expecting AUKCAR PhD students and early career researchers to engage with PPI has established a working mode that we hope will last. Regular interactions and proactive Patient Leads increase PPI network cohesion.

**Conclusion:**

With adaptation, the AUKCAR PPI model can be translated to international contexts.

## Background

### What is patient and public involvement?

The United Kingdom’s (UK) National Institute for Health Research (NIHR) was created in 2006 with the goal of “*conducting leading-edge research focused on the needs of patients and the public*”, with significant emphasis placed on the requirement to include patient and public involvement (PPI) in their funding programmes [1]. The establishment of INVOLVE, a NIHR organisation dedicated to promoting the inclusion of patient involvement in research, has enabled researchers to include PPI in their research [2]. The vision is research that is carried out ‘with’ or ‘by’ members of the public, rather than ‘on’ or ‘to’ them. Examples of PPI roles include membership of a patient advisory group, providing feedback on patient-facing resources or contributing to research priorities. This involvement is regarded as essential to ensure that the research is relevant to the population(s) it will impact.

### Why include PPI in research?

Involving patients and public members is not a new concept. Prior to the inception of the National Health Service, hospitals’ contributory schemes incorporated community participation in hospital governance [3]. Growing disillusionment in the 1950s led to health service user groups campaigning against poor quality healthcare – patient groups wanted better diagnosis, better treatment and for their voice to be involved in decisions about their condition. They challenged the viewpoint that clinicians and researchers knew best and were above reprehension for behaviours that were not deemed beneficial or acceptable by patients. Serious clinical failings, such as the Bristol Royal Infirmary and Alder Hey scandals, further strengthened the need to involve patient and public members and place them at the centre of healthcare [4].

Effective PPI contributes to the production of high quality research that is relevant and beneficial to patients, the public and the health service [5]. Increasingly, PPI is a pre-requisite in funding applications and in ethical reviews. However, there is still a gap between what is written in funding applications and what is needed to ensure that PPI is meaningful and patients are empowered to contribute. Concerns have been raised about the legitimacy of PPI in research, with criticisms of it being tokenistic and a ‘box-ticking’ exercise [6].

### What is stopping PPI from being part of research?

Barriers may be conceptual or practical. Despite commitment to collaborating with patients and the public in research, there is a lack of understanding from researchers regarding what PPI involves, how to support a diverse range of lay members, and the difference between PPI and qualitative research [7]. Traditional views of PPI in research have placed patients in a consulting role, where major decisions are made by academics and clinicians, rather than in a meaningful and engaged dialogue, collaborating from the outset and ensuring that the patient voice is heard throughout the research lifecycle [8]. Models and guides for involving patients in research exist, but the heterogeneity of research projects makes it difficult to determine a single ‘best’ approach [9]. Training is required to introduce researchers to the concept of involvement, the difference between involvement and participation, and how meaningfully to involve patient and public members in their projects.

**Fig. 1:**
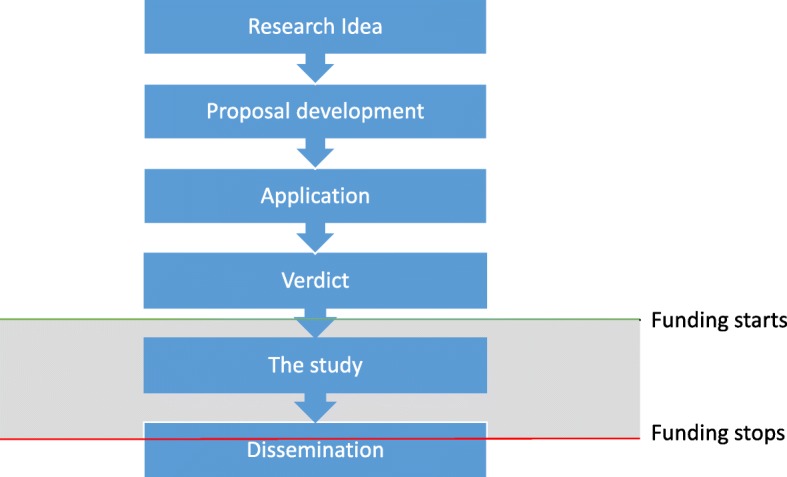
Project flow and timing of funding

Even researchers wanting to ensure meaningful involvement encounter a number of practical challenges, including funding, PPI recruitment and time constraints. The development of grant applications can be lengthy and involving patients incurs additional costs. Without funding, lay members may be bearing unacceptable out-of-pocket expenses. Although there are small bursary schemes available in England, money to support PPI typically only arrives after the successful securement of funding; yet, if lay members are only included when funding has been secured, the opportunity to contribute to the vital stage of research design has been missed (Fig. 1). Importantly, there is no PPI funding for unsuccessful grant applications to compensate lay members for their time. Similarly, it may be difficult supporting lay members in the dissemination of results when funding has stopped at the end of a grant.

From a PPI perspective, patients and the public are often unaware of opportunities to get involved with research, or are unsure about how to work with academics and clinicians. Lack of training and support can lead to uncertainty and anxiety about how to contribute or may cause concern about feeling overburdened by what is required [10]. Even though not every patient wants to be involved in healthcare research, barriers must be removed in order to support those who do [11]. PPI can be a daunting undertaking, pushing researchers out of their comfort zone to collaborate meaningfully with lay members. Academics and lay members both need support to fully and meaningfully engage with PPI.

## Moving from tokenistic PPI to meaningful involvement

The Public Involvement Impact Assessment Framework was developed to measure the impact of PPI on research [12]. However, measuring impact by evaluating PPI as an intervention is inadequate as involvement needs to be thought about as “*conversations that support two-way learning*” [13]; therefore, only measuring PPI impact on research without considering the impact on involved patients omits half of this equation. This highlights the need for guidelines that offer advice about objective measurement of meaningful PPI involvement, so that researchers know if they have incorporated PPI in a sound way.

In 2018, the NIHR, the Chief Scientist’s Office of the Scottish Government, Health and Care Research Wales and the Northern Ireland Public Health Agency together launched the National Standards for Public Involvement [14]. These standards were the result of a public consultation that garnered over 700 responses. There are six overarching standards, each with multiple indicators, as follows:

- Inclusive opportunities
- Working together
- Support and learning
- Communications
- Impact
- Governance

The standards can be used to guide researchers to meaningfully engage in PPI as well as to highlight areas for improvement and to measure the impact of PPI research and its research outputs.

### PPI at the heart of everything we do: an exemplar

We reflect here on our experience of delivering PPI within the context of the Asthma UK Centre for Applied Research (AUKCAR) [15]. AUKCAR is a UK-wide virtual centre bringing together world-leading academics and internationally respected clinicians to collaborate on key areas of research to improve the management of asthma [15]. From inception, AUKCAR’s vision has been to keep patients and public at the heart of all we do, aligned with the aims of the charity (and our funder) – Asthma UK. Due to AUKCAR’s foundational work and experience, we were one of 10 UK sites chosen to pilot the workability of the NIHR UK Standards for Public Involvement at 5 years after inception [14].

#### A strong PPI organisational infrastructure

A supportive PPI organisational infrastructure was planned, itemised as part of the initial AUKCAR budget and implemented during the formative years of the Centre. The AUKCAR PPI team has four patients as PPI Leads (three of whom are grant holders), a designated senior academic Lead, and a funded research fellow responsible for co-developing and spreading a common set of PPI norms, facilitating PPI activities and coordinating the Patient Advisory Group – our volunteer group of over 100 people impacted by asthma. To ensure long-term embedding of PPI, grant applications for individual projects include a budget to contribute to PPI within the Centre. The hope was that this arrangement would enable us to sustain the AUKCAR PPI infrastructure to support PPI at the pre-funding and dissemination stages (Fig. 1). Whilst this is realistic in the context of large trial and programme grants, small grants and PhD fellowships rarely have enough budget to make this a viable model and on-going infrastructure funding has proved to be invaluable.

#### The key role of the PPI research fellow

The PPI research fellow is a single-point of contact for both researchers and PPI colleagues, developing strong relationships with lay members, providing consistency, and cascading down PPI norms and values. All PPI activity goes through the research fellow, who oversees the amount of work sent to individual PPI colleagues, making sure no one is overburdened or overlooked.

Researchers contact the PPI research fellow to discuss their projects and PPI requirements, ideally at planning stage. Study details and the lay summary are emailed to all PPI colleagues by the research fellow and PPI colleagues chose when, in what and how they want to get involved, based on interest, skills and availability. AUKCAR PPI colleagues are not required to commit to a specific contribution when joining the Patient Advisory Group, all levels of involvement are welcome. Involvement opportunities over the last 5 years have included reviewing funding applications, providing feedback on patient-facing documents, inputting on website design, research steering group membership, co-application on project grants, co-authoring research articles, and presenting at conferences.

#### Building and strengthening the AUKCAR Patient Advisory Group

Initially, the Patient Advisory Group was established by advertising and recruiting lay members from asthma clinics and existing involvement groups. Three PPI Patient Leads, known to researchers from existing projects, joined the team as grant holders from the start, with a fourth lead emerging naturally from the Patient Advisory Group over time. The PPI organisational infrastructure we developed includes Research Leads, PPI Research Fellows and Patient Leads to facilitate the sharing of norms and values, which, over time, increase the strength and cohesion of the group.

Technology, the web and social media facilitate the process of network building, enabling members to create online contacts and stay in touch at virtually no financial cost. We have increased the accessibility of our documents for PPI colleagues who have visual impairments, but we are aware that communicating mostly by email or phone does exclude some members of the public. The AUKCAR Patient Advisory Group fosters peer support between its members (both online and offline), which has been welcomed by many members, who report that being part of the group had a positive impact on their lives.

Benefits of being part of the AUKCAR PPI community is discussed by one of our Patient Leads:

“*The positive impact for me is it has changed a situation where asthma was taking everything away, to one where I can do something productive despite having asthma and hopefully make a difference for those with asthma in the future.*”

The strength of our Patient Advisory Group increasingly lies in the links created by Patient Leads. As co-applicants on AUKCAR’s grants and members of the Centre Management Committee, they have a good understanding of the research environment and have progressively acquired authority within the organisation through their regular contributions. The confidence that PPI colleagues increasingly acquire through being members of a group gives them a unique and strong voice. We are witnessing PPI shaping research, even from the very early stage of co-creating the research questions. For example, one of AUKCAR’s PhD students is working on a project that evolved directly from a PPI member’s suggestion.

#### PPI and the AUKCAR post-graduate scheme

In addition to collaborating on research projects, PPI colleagues have a role as educators. They provide knowledge about asthma to researchers who have no direct experience of living with the condition. Successful strategies for enhancing the sharing of expertise have included joint training courses and informal discussions between research and PPI teams.

PPI colleagues have proved invaluable to AUKCAR’s postgraduate training scheme, which aims to nurture PhD students to become the next generation of leaders in world-class asthma research. PPI colleagues are involved in reviewing PhD proposals and candidate applications, and sit on the interview panels. Integrating PPI at all stages of research is a requirement in all AUKCAR PhD projects. Training in working with PPI colleagues and support with facilitating involvement is provided by the research fellow, free to PhD students, who inevitably work on a limited research budget. Enabled by our infrastructure funding, AUKCAR PhD students learn first-hand about the benefits of working with PPI colleagues and integrate descriptions of their PPI work into their publications and theses. Subsequently, we have developed a tranche of early career researchers for whom PPI is the norm.

The value of involving PPI colleagues in AUKCAR’s Postgraduate training scheme is clear from one student’s personal experience:

“*When I began my PhD, I had little understanding of PPI, and struggled to see how it was relevant to my quantitative project. While the support from the PPI research fellow has been instrumental in facilitating work with PPI members, it has been the numerous interactions with the PPI members themselves that have strengthened my understanding the most. I feel fortunate to have been part of a research group with an existing Patient Advisory Group as this allowed me to realise the benefit of PPI early in my PhD, while it has the greatest potential to impact my project.*”

Both formal and informal training opportunities are available to PhD students (and to all AUKCAR researchers) such as webinars on PPI-specific topics and opportunities to pilot research methodologies with PPI colleagues. We are currently developing training modules for PPI colleagues to ensure that they are fully supported in the research environment.

#### A central role for PPI at the AUKCAR annual scientific meeting

PPI colleagues have an active role in our Annual Scientific Meeting, including reviewing abstracts, presenting research, acting as panel members, networking with researchers and judging presentations. The cohesive network, mutual support and familiarity with both the research and the researchers has enabled PPI colleagues to actively participate in presentations, involve themselves in discussions with other stakeholders and help with the effective dissemination of AUKCAR research through media channels [16]. Networking between researchers and PPI colleagues is an essential catalyst of new and valid research questions and has resulted in the development of projects that are grounded in the real world.

#### Evaluating our PPI

In 2018, the invitation to be a test-bed site for the six NIHR UK Standards for Public Involvement in Research provided us with the opportunity to evaluate the impact of PPI on AUKCAR research, researchers and lay volunteers. Working as a team comprised of academics and PPI colleagues, we used the six standards to document the domains where we had achieved meaningful involvement and to identify areas for improvement (Table 1). Now in our sixth year as AUKCAR, we have incorporated the standards within our PPI strategy, applying them to all our work going forward. We have adopted a pragmatic approach ensuring that individual projects incorporate tailored PPI involvement, methods and philosophies, whilst avoiding the pitfall of rigid ‘box ticking’.

**Table 1:** Application of NIHR UK Standards for Public Involvement in Research in AUKCAR

UK Standard for Public involvement in Research	Achievement Status	Examples of how we achieved this	Areas for further improvement
**Inclusive opportunities:** Offer public involvement opportunities that are accessible and that reach people and groups according to research needs	Achieved	The research fellow keeps in touch with the Patient Advisory Group and advertises opportunities by email and social media. We work on a first come first served basis; PPI colleagues choose what they want to be involved in, when and how.	Consider sharing information via radio or through leafleting/advertising in community spaces
**Communications:** Use plain language for well-timed and relevant communications, as part of involvement plans and activities	Achieved	In conjunction with PPI colleagues, we produced “PPI request forms” for researchers seeking advice. This ensures requests contain all information PPI colleagues may want to know in order to commit to involvement in a project.	Develop a communication plan for involvement activities
**Support and learning:** Offer and promote support and learning opportunities that build confidence and skills for public involvement in research	Partially achieved	We regularly provide opportunities (e.g. monthly meetings) for PPI colleagues to learn and contribute to AUKCAR ongoing research projects, explaining research methods and their implementation in lay language, building their skills and confidence in co-developing the AUKCAR research.	Provide online training on how to be involved for researchers and PPI colleagues. This is currently in production.
**Working together:** Work together in a way that values all contributions, and that builds and sustains mutually respectful and productive relationships	Achieved	The AUKCAR PPI strategy was co-produced with PPI (available on our website). The quarterly meetings, with the PPI Leads and PPI Research Fellow, provide opportunities to collaborate and develop research initiatives.	More training for researchers about PPI and available AUKCAR support, including training on ‘soft’ skills such as group facilitation
**Governance:** Involve the public in research management, regulation, leadership and decision making	Achieved	We have four Patient Leads within AUKCAR who are grant-holders. and attend Centre Management Committee meetings	There is a need to identify external sources of funding to make AUKCAR PPI sustainable in the long-term.
**Impact:** Seek improvement, by identifying and sharing the difference that public involvement makes to research	Achieved	Projects hold regular meetings with PPI colleagues to provide update on project status, gather feedback and show the impact that PPI contribution has had on the project. We deliver this in a format of “you said …, we did …”	Potential for annual survey of researchers and lay members to gather feedback on PPI. We will be introducing quarterly newsletters for larger projects to keep members regularly engaged

### What were the lessons learned?

A properly funded supportive organisational infrastructure is integral to implementing meaningful PPI and reducing unintentional tokenism. This needs to include a person (a research fellow in our model) able to provide advice and guidance to researchers, assist in tailoring an involvement plan to the specific needs of each project, and (crucially) available throughout the research lifecycle. Recruiting and involving PPI colleagues is time consuming and the presence of a dedicated person and supportive infrastructure minimises the burden for researchers, making consistent PPI (relatively) easy and thus more likely to happen. An established Patient Advisory Group reduces the time required for PPI recruitment for individual projects and ensures PPI inclusion in the research design from the pre-funding and dissemination stages.

The collaboration with senior academics is strengthened by Patient Leads sitting on the Centre Management Committee and co-developing the vision and research strategies of AUKCAR. Patient Leads are mentors to members of our Patient Advisory Group, leading by example, being a contact point for queries, and supporting and instilling confidence in less experienced members. Training and support allows our lay members to grow in their roles and we are increasingly looking to formalise our PPI training offerings. Communicating effectively throughout their involvement activities reinforces their perceived role as collaborators with power to influence research decisions. Most importantly, PPI members are valued colleagues who have a legitimate place at the decision table.

The participation of PPI colleagues is largely through email and telephone, allowing flexibility around members’ individual circumstances. Technology is an additional important enabler. Emails and social media platforms increasingly allow people to create links that strengthen the Patient Advisory Group and to easily maintain contact. PPI colleagues appreciate that the commitment is flexible, overcoming a barrier they have found when attempting to join other patient groups.

## Global translation – PPI beyond the UK

Having learnt from the development of a successful model within AUKCAR, we are working together with researchers in an NIHR Global Health Research Unit on Respiratory Health (RESPIRE) [17] to adapt this involvement model to different cultural contexts. Similarities with the AUKCAR model include mandatory inclusion of PPI plans in RESPIRE projects and a funded PPI research fellow based in the UK providing support and guidance to RESPIRE researchers to enable the meaningful involvement of patient and community members. However, we are learning that there are factors that are not transferable.

PPI in health is a global mandate [18] and its impact in high-income countries is increasing [19, 20]. Yet, this is not the case for low- and middle-income countries (LMIC) where PPI in health research is unusual [21]. There are benefits and challenges to incorporating PPI in LMICs – PPI can help in the identification and prioritisation of research topics to those relevant to end-users, assist in the practical details of data collection, and help in the interpretation and implementation of findings in communities where traditional health practices may be the norm and health literacy levels may be limited [5]. Barriers to PPI, such as a lack of understanding of the underlying concept by both researchers and the public, variations in infrastructure and cultural norms, power relationships between researchers and the researched, and resource limitations are the major challenges faced in developing PPI in LMICs [21]. We are finding it helpful to engage with the public as a community rather than individually and to use alternative terminology such as ‘community engagement and involvement’.

An important aspect of PPI is to address global inequity in health research. Equal partnerships and collaborations with patients and the public can improve empowerment, ownership, sustainability and research capacity in LMICs [22]. This will require the development of strategic frameworks to implement the policies and build the organisational infrastructure needed for the advancement of PPI in LMICs. To this end, RESPIRE is funding a research fellowship for a senior colleague to understand and develop PPI in research in Malaysia and potentially provide knowledge that can be more widely applied in LMICs.

## Conclusions

Our approach to a PPI organisational infrastructure has addressed many key PPI challenges, helping to overcome barriers inherent in a system that relies on project funding by ensuring that support is available to include PPI throughout the lifetime of a project, from early ideas to final dissemination. The provision of a supportive environment as well as specifically dedicated personnel has allowed effective sharing and spreading of a common set of PPI norms and values, with the aim ‘keeping patients and the public at the heart of everything we do’. Communication technology advances, along with opportunities for face-to-face meetings, have been instrumental in creating a cohesive Patient Advisory Group. Cultural differences between the UK and other countries require the tailoring of PPI needs to individual country settings to ensure that research continues to be based on real-life circumstances and that it positively impacts on the lives of people living with health conditions worldwide.

## Data Availability

Not applicable.
